# Epoxy\Epoxy Composite\Epoxy Hybrid Composite Coatings for Tribological Applications—A Review

**DOI:** 10.3390/polym13020179

**Published:** 2021-01-06

**Authors:** M. M. A. Baig, M. Abdul Samad

**Affiliations:** Department of Mechanical Engineering, King Fahd University of Petroleum and Minerals, Dhahran 31261, Saudi Arabia; mmurtuza@kfupm.edu.sa

**Keywords:** epoxy coatings, tribology, friction, wear

## Abstract

Epoxy composite coating systems generally find their usage in applications such as, fluid handling systems to protect components from corrosive media. However, their use in demanding tribological applications such as, in sliding components of machines, are known to be limited. This is often attributed to their low load bearing capacity combined with poor thermal stability under severe *p*-*v* regimes. Researchers have tried to enhance the tribological properties of the epoxy coatings using a combination of several types of micro/nano sized fillers to produce composite or hybrid composite coatings. Hence, this review paper aims to focus on the recent advances made in developing the epoxy coating systems. Special attention would be paid to the types and properties of nano-fillers that have been commonly used to develop these coatings, different dispersion techniques adopted and the effects that each of these fillers (and their combinations) have on the tribological properties of these coatings.

## 1. Introduction

**Figure 1:**
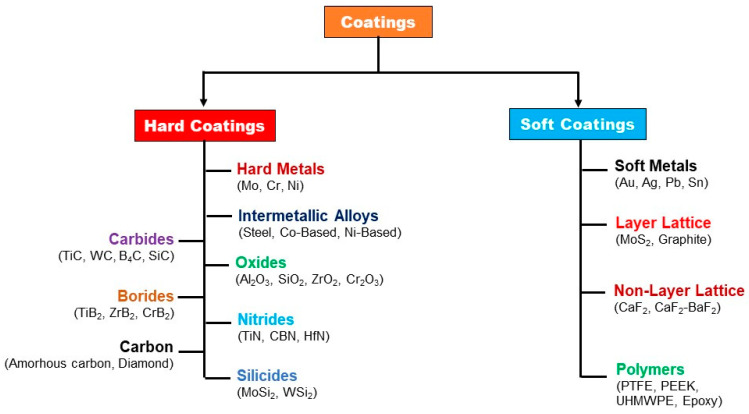
Classification of different types of coatings.

Coatings in general, have primarily been used for protecting components subjected to corrosion, various forms of wear such as abrasion, and in some cases, for decorative purposes [1]. They find their use in a wide spectrum of applications in oil and gas, petrochemical, marine [2,3], construction [4], medical [5], defense, paper, power, metal processing and cement industries [6]. Figure 1, shows the broad classification of coatings into hard and soft coatings along with their sub-categories.

Recently, soft polymer coatings have attracted the attention of researchers because of their ease of fabrication, excellent tribological properties and better adhesion to the substrates. As can be seen from Table 1, epoxy resins have good shear strength as compared to some commonly used polymers. Epoxy resins also show lower curing shrinkage, better thermal stability and good mechanical strength as compared to other resins as shown in Table 1. In addition to that, its chemical inertness and good corrosion resistance makes it an attractive candidate for the coating systems [7,8,9,10]. Despite of the above mentioned properties of epoxy coatings, their use in various demanding tribological environments are known to be limited. This is often attributed to their low load bearing capacity combined with poor thermal stability under severe *p-v* regimes. Researchers in both academia and industry have tried to enhance the tribological properties of the pristine epoxy matrix using a combination of several types of micro/nano sized fillers to produce composite coatings. Hence, the focus of this review paper is to comprehensively summarize the types and properties of nano-fillers that have been commonly used to develop epoxy composite coatings and the effects that each of these fillers (and their combinations) have on the tribological properties of the coating. Most importantly, this paper aims to identify the significant gaps existing in the literature and recommend directions for future research works in the field of liquid and powder epoxy coatings.

## 2. Properties of Epoxy Resins

Epoxy resins are generally classified into three types as bisphenol-A, bisphenol-F and novolac [6,13]. Bisphenol-A is formulated by reacting phenols with acetone while the rest are formulated by reacting phenols with formaldehyde. Excess phenol is required in the synthesis of novolac. These are finally cured after reacting with epichlorohydrin. Novolac epoxy resins are modifications of bisphenol-F resins produced by reacting excess phenol and formaldehyde. Among these resins, bisphenol-A is most commonly used for the coating systems due to its good adhesion and good chemical and wear resistance [13,14].

It is important to note that in addition to the base resins used, the solidifier components, which are primary, secondary or tertiary amine compounds, also play a very important role in determining the properties of the cured system. The properties of base and solidifier components, typically used in the formulation of epoxies are summarized in Table 2 [6].

Regardless of the aforementioned properties of pristine epoxies, its application has been limited due to their, low fracture toughness, low impact resistance, and inadequate tribological performance.

## 3. Modification of Epoxy Resins for Improved Properties

Few attempts were made to modify the pristine epoxy resin to improve its properties by adding polymers, or fluorinated and siloxane compounds. Fluorinated and siloxane compounds are well-known for imparting good tribological properties to coatings [15,16]. Abrasive wear resistance of UV curable epoxy acrylate formulations was improved when modified with the (0–0.8 wt%) reactive fluorinated siloxane copolymer [15]. The wear resistance further increased with increasing content of the modifier due to anti-friction characteristics of fluorinated and siloxane compounds present on the surface of modified coatings. In another study, the epoxy modified with silicon-containing polyepoxies and bioactive coordination compounds showed enhanced anti friction and scratch resistance [17]. The tribological properties of epoxy resin coatings are also affected by the molecular structure of curing agents. Dan Liu et al. [18] used three types of curing agents such as, the chain-shaped diethylenetriamine (DETA), ring with side-chain shaped isophorone diamine (IPDA)and benzene-shaped m-phenylenediamine (m-PDA), respectively, to cure epoxy resin coatings. The linear and flexible chain structure of DETA promoted the sliding process resulting in lowest friction coefficient. However, the epoxy coatings cured with benzene-shaped m-PDA resulted in highest friction coefficient.

## 4. Types of Epoxy Composite Coating Systems

More recently, researchers also started modifying the pristine epoxies using a wide assortment of micro/nano sized fillers to produce stand-alone or hybrid micro/nanocomposite coatings to overcome the various limitations of pristine epoxy systems.

The epoxy coating systems can be classified as liquid based and powder based, which is also referred to as fusion bonded epoxy (FBE) coating system. In the liquid based epoxy (LBE) coating systems, the starting epoxy matrix resin is in liquid form while in FBE system the starting epoxy matrix resin is in powder form.

### 4.1. Fillers Used in LBE Coating Systems

**Figure 2:**
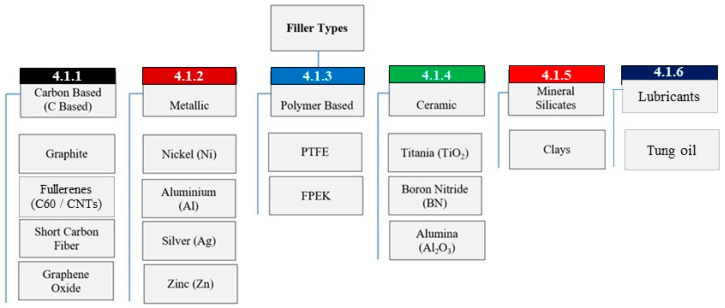
Classification of different fillers used to reinforce epoxy matrix.

In the liquid based epoxy (LBE) coating systems, the starting epoxy matrix resin is in liquid form. Various types of nano-fillers have been extensively used to improve friction, wear and load bearing properties of pristine epoxy resins. Figure 2 presents a summary of commonly used fillers added, to modify liquid epoxy resin systems.

As can be observed, fillers are broadly classified into, carbon-based, metallic, polymer based, ceramic, mineral silicates and lubricant fillers. These fillers can be used to reinforce the epoxy matrix individually or in combination of two or more. When a combination of several types of nano-fillers are added together, the system is often referred to as a hybrid system. Hybrid systems have been proven to deliver the best properties of each of the fillers when added in specific compositions determined through a series of iterative experiments. Whether added as a stand-alone or as a hybrid, different nano-fillers have resulted in a significant improvement of the tribological properties in terms of lowering of coefficient of friction and lowering of wear of the mating interfaces under sliding conditions. The following sections presents the role played by each of these reinforcements in improving the properties of epoxy coating systems.

#### 4.1.1. Carbon Based Fillers

It has been reported [19,20,21,22,23] in several studies that carbon fillers in polymer matrices in general and in epoxy matrix in particular could reduce the friction of the composites due to their self-lubricating effect.

- *Graphite:* Graphite, an allotrope of carbon with multi-layer of carbon atoms possess low shear strength due to the weak Vander Walls forces between adjacent layers [24]. Studies have reported that graphite when used as a solid lubricating filler in the fabrication of epoxy composite coatings resulted in reduced friction and wear rates [25,26].
- *Graphene:* Graphene is a monolayer of carbon atoms arranged in hexagonal honeycomb structure. Graphene and its derivative, graphene oxide are commonly used carbon based fillers owing to their outstanding thermal, physical, optical, electrical, mechanical and tribological properties [27,28,29,30,31,32,33,34,35,36,37,38,39,40]. The weak Vander Walls forces between the intermediate layers of a typical lamellar structure of graphene, provides exceptional shear slide capability [29,37,39,41,42,43,44]. This solid lubrication property of graphene and its derivatives has drawn much attention in the fabrication of anti-friction and anti-wear epoxy coatings [45,46,47,48,49,50,51]. Some researchers have non-covalently functionalized graphene [39,52] and graphene oxide [37,53] to improve their dispersion and interfacial bonding with epoxy matrix. These fillers are also an ideal support for synthesizing nano-particles in the fabrication of hybrid coatings [45,54,55,56]. Thereby, agglomeration of secondary nano-fillers and the stacking of graphene and graphene oxide can be avoided.
- *Fullerenes:* Allotropes of carbon in spherical and tubular shapes are called Fullerenes. The most abundant member of spherical Fullerene family is C60 which is also called bucky ball [6,30]. They possess high compressive strength and high electron affinity, which can be exploited to increase their functionalities [30]. Fullerenes in the shape of tubes, are called carbon nano tubes (CNTs). Besides large surface area, CNTs possess unique structure combined with outstanding properties, such as, high elastic modulus, high tensile strength, high electrical and thermal conductivity, and excellent tribological properties [23,31,32,57,58,59,60]. A single graphite layer forming cylindrical shape constitutes a single-walled CNT while many concentric graphite layers constitutes multi-walled CNT (MWCNT). A strong covalent bond exists between atoms within each graphite layer. The adjacent layers held by weak van der Waals forces provide exceptional shear slide capability reducing friction [61]. The surfaces of these layers serve as a potent support for growing other nano-particles during the fabrication of hybrid coatings [62].
- *Carbon Nano Fibers:* Carbon nano fiber (CNF) features high modulus and strength, large specific surface areas and outstanding friction and wear performance [63,64,65,66]. Their surface, serves as a potent breeding place for secondary reinforcements during the fabrication of hybrid filler material.

#### 4.1.2. Metallic Fillers

Metallic materials are basically used as reinforcements in polymer matrices to improve electrical, thermal, magnetic and mechanical properties. A comparative study of tribological properties of epoxy coatings, reinforced with metallic micro-fillers, such as, nickel (flakes form), aluminum (atomized), silver (irregular shaped) and zinc (irregular and spherical shaped) was carried out by Browstow et al. [67]. The addition of aluminum fillers resulted in the lowest coefficient of friction and wear when cured at lower temperatures owing to their spherical morphology resulting in rolling friction between the contacting surfaces during the ball-on-disk test. In another study, Mn-Zn Ferrite particles were used as fillers to fabricate electromagnetic absorbing epoxy composite coating. The surface of the fillers was modified with Oleic acid to hinder the possible agglomeration of nano-particles due to magnetic attraction between particles [68,69,70,71]. Transition metal sulphides such as, MoS_2_, Ag_2_S, ZnS, NbSe_2_ have been used as solid metallic lubricative fillers. To avoid the agglomeration of MoS_2_ nano-sheets and ZnS nano-particles, they were grown on the surfaces of primary fillers by hydro-thermal method. MoS_2_ nano-sheets were synthesized on the surfaces of CNTs [62], h-BN [72], Carbon nano fibers [63], and ZnS nano-particles were loaded on the surfaces of CNTs [57], GO [45] to fabricate epoxy hybrid composite coatings. Recently, ternary hybrid material was synthesized by hydrothermal method where both CNTs and MoS_2_ were grown on the surface of GO [62]. To inhibit the grain growth and agglomeration, Ag_2_S nano-particles were in situ synthesized during curing reaction of epoxy hybrid nanocomposite coatings by Pyrolysis reaction [53]. The NbSe_2_ filler was reported to improve wear resistance and anti-friction properties of epoxy nanocomposite coatings [73].

#### 4.1.3. Polymer-Based Fillers

The commonly used polymer based fillers are fluorinated polymers such as polytetrafluoroethylene (PTFE) and fluorinated poly (aryl ether ketone) (FPEK). These are low friction hydrophobic materials due to their low surface energies [37,67,74,75]. However, their high wear rate is overcome by incorporating a second reinforcement like graphene [37] or Ag_2_S [53]. Brostow et al. [67] evaluated the effect of different secondary metallic reinforcements of different shapes with FPEK as a primary filler, on the tribological properties of epoxy coatings. It was observed that the addition of aluminum fillers resulted in the lowest coefficient of friction and wear. The waste tire rubber (Styrene-Butadiene Rubber)-an elastomeric polymer, was recently used as filler in the fabrication of cost-effective composite epoxy coating. The coating displayed better wear resistance and anti-friction properties [76].

#### 4.1.4. Ceramics

Metal oxides, carbides, nitrides, borides, silicides, etc. are referred to as ceramics [6]. The covalent functionalization by hydroxylation was reported to improve dispersion of inorganic fillers such as, HBN [72], Ti_3_C_2_ [41] in organic epoxy matrix with strong interfacial interaction. However, the covalent functionalization of SiC [77], CBN, and HBN [78] using inorganic-organic amino compounds was reported to have an additional advantage of promoting adhesion between the coating and the substrate. Ceramic fillers are expected to enhance the tribological properties of the epoxy coatings. The incorporation of micro-SiC [77] and nano-SiO_2_ [79] have resulted in reduction in friction coefficient, but with an increase in wear rate. A general reduction in wear and friction was recorded with Ti_3_C_2_ [41,80], CBN [78], and HBN [72,78] filled nanocomposite coatings. The maximum reduction in friction was obtained when HBN was used as filler, while the composite coating reinforced with CBN displayed the highest wear resistance [78]. Karasinski et al. [81] presented a comparative study on scratch resistance of the epoxy composite coating filled with nano metal oxides (ZnO, Al_2_O_3_). The incorporation of Al_2_O_3_ resulted in improved scratch resistance as compared to the neat epoxy coating and the epoxy coating reinforced with ZnO, since ZnO had a catalytic effect on the curing reaction contributing to the brittle behavior of the nanocomposite.

#### 4.1.5. Mineral Silicate

Katiyar et al. [26] reported improved friction and wear properties when talc i.e., hydrous magnesium silicate was used in the fabrication of epoxy hybrid composite coating. In another study, the inclusion of magnesium silicate had adverse effect on the cavitation erosion wear resistance of the coating due to increased number of pores which acted as nucleation site for cracks [82].

#### 4.1.6. Lubricating Fillers

Considering the self-healing and self-lubricating properties of tung oil, epoxy coatings were fabricated incorporating polysulfone microcapsules containing tung oil. Polysulfone was used as encapsulating material due to its high mechanical strength and outstanding physical, thermal, and chemical stability. A significant reduction in wear rate, friction coefficient, and corrosion rate was reported for the composite coating. The improved self-lubricating and anti-corrosion performance of the composite coating was attributed to the formation of a transfer film [83].

### 4.2. Dispersion Techniques Used in LBE Coating Systems

Uniform dispersion of fillers in the epoxy matrix is crucial in fabricating wear resistant coatings with reduced friction. A variety of dispersion techniques used by researchers includes stirring, sonication, extrusion, and blending. Sonication has been the most common dispersion technique adopted by many researchers in the fabrication of non-hybrid [23,30,39,52,68,77,78,80,84] and hybrid [26,37,57,62,63,72,85] epoxy composite coatings. The success of this technique is attributed to the high frequency ultrasonic waves, which promote uniform dispersion and de-agglomeration of fillers. Table 3 summarizes the different dispersion techniques adopted by researchers to uniformly disperse different fillers in the epoxy matrix.

As can be observed from Table 3, in one instance when the resin was in the form of pellets, an extruder was used for melting and dispersing the fillers. In another instance, ball milling was used for dispersing of pre-treated CNTs as it was observed that the high stress field in sonication could result in the fracture of CNTs. However, sonication was the most preferred dispersion technique adopted for dispersing different fillers effectively, in the epoxy matrix.

### 4.3. Coating Techniques Used in LBE Coating Systems

Among various techniques adopted by researchers, wire bar coating [23,30,52,76,78] and air-spray coating using spray gun [41,45,53,57,62,63,72,85] have been the most commonly used techniques to coat the substrate with non-hybrid and hybrid epoxy composite coatings respectively. The coating technique to be used depends largely on the application feasibility such as the geometry of the substrate to be coated, coating type, resin viscosity etc. and can vary widely between laboratory and industrial settings.

It is also to be noted that the longevity of the coating depends upon its adhesion to the substrate [6]. Pre-treatment techniques like air-pyrolysis [85] and oxygen-plasma [6,86] have come into existence to improve the adhesive strength. Katiyar et al. [26] improved the surface energy of the substrate by generating hydroxyl functional groups over it by oxygen plasma treatment. In some studies, sand blasting [53] and abrasive (Al_2_O_3_) blasting [85] was used to create anchoring profile on the substrate, which promotes mechanical adhesion. The coating substrates are decontaminated by cleansing with Acetone or Ethyl Alcohol prior to depositing the coating on them.

The coating techniques adopted by various researchers along with different surface pre-treatments are summarized in Table 4.

It can be observed from Table 4 that researchers have commonly used air-spray and wire bar coating techniques for depositing epoxy coatings on various substrates. The coating thicknesses in the range of 21.2–80 µm, 15–80 µm, and 2–300 µm were achieved by the spin, air-spray, and wire bar coating techniques respectively, indicating that the wire bar coating emerges to be the most scalable coating technique. However, the wire bar coatings were observed to have the slowest while the spin coatings had the fastest curing rate.

### 4.4. Tribological Properties of LBE Coating Systems

In this section, an extensive review is presented about the tribological performance of the epoxy coatings. For clarity purpose, initially the tribological performance of non-hybrid composite coatings are discussed followed by the tribological performance of the hybrid composite coatings.

#### 4.4.1. Non-Hybrid LBE Composite Coatings

A non-hybrid epoxy composite coating in this review is defined as the one, which has only one filler added to the pristine epoxy matrix. Hence, this section reviews the effects of these different types of individual fillers on the tribological performance of the epoxy coatings.

Graphene has been an attractive filler for wear resistant coatings owing to its lubricating effect and outstanding shear-slide capability. However, its hydrophobicity hinders its uniform dispersion in water-borne epoxy systems. Hence, researchers have tried to functionalize the graphene surface with a diazonium salt with hydroxyphenyl groups to obtain uniform dispersion in the epoxy matrix [39]. The hydroxyl groups grafted on graphene surface further forms a stable cross-linked network with epoxy resins upon curing. Pristine epoxy coatings and the composite coatings were deposited using spray gun on stainless steel substrates. Frictional response against steel counter face under dry and lubricated condition was evaluated using a ball-on-disk configuration. Compared to pristine epoxy coatings, the composite coating filled separately with graphene oxide and functionalized graphene reduced the friction coefficient by 15 and 37 times, respectively. Relative wear life of different coatings in terms of coating worn-out time against rotating nylon ring was also determined. The functionalized graphene filled epoxy composite coatings had demonstrated a longer wear life compared to that filled with graphene oxide. The improved tribological properties were observed due to strong bonding between graphene and epoxy binder promoted by functionalization. Moreover, the wear life increased with increasing size of functionalized graphene sheets owing to enhanced self-lubricating effect.

To overcome the challenge of agglomeration of graphene in the epoxy matrix, Chen et al. [52] non-covalently functionalized nano-Graphene sheets by reacting it with poly(2-butylaniline) in organic solvent. The functionalized Graphene sheets were added to epoxy matrix and amine hardener. Addition of small amount (0.5–1 wt%) of functionalized graphene sheets demonstrated an improved corrosion and tribological performance. An optimum reduction in wear rate and friction coefficient by 85% and 17% respectively was reported with the incorporation of 0.5 wt% of functionalized nano-graphene sheets in epoxy matrix.

Le at al [61] used CNTs as fillers in commercial epoxy primer. To ensure uniform dispersion of CNTs, environment friendly ball milling was used for mixing. The friction behavior was studied under constant and ramping load using reciprocating sliding ball-on-plate test rig. A slightly lower friction coefficient was recorded with the inclusion of CNT as filler. Though none of the coatings failed after 16 cycles (2000 s) of testing under 1.2 N load, epoxy composite coatings featured smooth wear marks indicative of enhanced wear resistance. Multiwalled carbon nano tubes (MWCNT) were also found to have a profound effect on the mechanical, tribological and corrosion properties of the epoxy coatings. Khun et al. [23] explored the effect of varying concentration of MWCNTs filler on the mechanical, tribological, and corrosion properties of the epoxy composite coatings. The adhesive strength not only improved, but also increased with increasing content of MWCNT filler (0, 0.1, 0.5 wt%) as revealed by atomic force microscopy and blister test. Friction and wear tests were performed on ball-on-disk micro tribometer against steel ball counter face. Though the friction coefficient was observed to reduce with increasing content of MWCNT filler, the width and depth of wear tracks on the coatings were too small to be measured. The improvement in wear resistance was indirectly inferred through the increase in young’s modulus with increasing content of MWCNT filler. The improved tribological performance of the composite coating was attributed to the increased rolling and solid lubricating effects of MWCNTs together with enhanced load bearing capacity. Moreover, increasing content of MWCNTs also resulted in improved corrosion resistance of epoxy composite coating.

Besides filler content, filler shapes were also found to effect tribological properties. The effect of filler shapes was evaluated by incorporating (0–1 wt%) functionalized fullerene C60 and (0–1 wt%) functionalized graphene as nano-fillers into epoxy based coatings [30]. The dispersion of C60 and graphene in epoxy resin was improved by modifying them with a silane coupling agent 3-aminopropyltriethoxysine. The results of progressive load scratch test revealed an improvement in scratch resistance of the composite coating. Ball-on-plate tribo-tests were performed against steel ball under dry and sea water condition. A progressive reduction in friction coefficient and wear rate with an increase in filler content from 0 to 0.5 wt% was reported. A further increase in filler content deteriorated the friction and wear performance due to agglomeration. The epoxy composite coating reinforced with modified fullerene C60 showed better scratch and wear performance, but poor corrosion performance in comparison to modified graphene reinforced coating. The spherical shape of C60 molecule not only contributed to increased load bearing capacity of the coating, but also presented easier permeation of corrosive solutions due to its relatively smaller surface area.

The findings of Aviles et al. [84] showed that graphene dispersed in ionic liquids improved the tribological properties of epoxy composite coatings. Mild steel substrates were single- and double-layer spin coated with epoxy resin reinforced with modified 0.1 wt% graphene dispersed in 9 wt% ionic liquids. The ionic liquids used were apriotic (1-octyl-3-methylimidazolium tetrafluoroborate) and priotic bis (2-hydroxyethyl) ammonium oleate). Pin-on-disk sliding wear tests were performed against AISI 316L to evaluate the tribological performance of the composite coatings. An effective reduction in friction coefficient by 70% with no sign of surface wear was achieved with a single layer coating of epoxy resin modified with 0.1 wt% graphene dispersed in apriotic liquid.

Wang et al. [68] developed electromagnetic coatings by reinforcing epoxy resin with oleic acid modified Mn-Zn ferrite particles. The addition of non-modified filler was reported to increase the roughness and friction coefficient. The addition of surface modified filler with oleic acid presented the friction coefficient comparable to neat epoxy coatings, but with better stability. This was attributed to the uniform dispersion of the fillers in the epoxy resin and improved adhesion of the coating to the substrate because of the surface modification.

Yingke Kang et al. [79] used nano-SiO_2_ surface-capped with epoxide as a filler in epoxy resin to fabricate epoxy composite coatings. Effect of filler content and the normal load on friction and wear characteristics of the coating were evaluated using ball-on-plate dry sliding wear test against a steel counter face. The coatings with lower content of filler resulted in smaller surface roughness and reduced friction coefficient. Higher content of nano-silica filler adversely affected the surface roughness and the friction coefficient due to possible aggregate formation. On the contrary, lower contents of the filler increased the wear rate while the higher contents yielded wear rates close but not better than that of neat epoxy. At higher contents of abrasive nano-fillers, more severe aggregate formation on the coating surface aided in resisting wear by the counter face.

Yan Hao et al. [77] fabricated epoxy composite coatings reinforced with 50 wt% of functionalized SiC particles. Sliding wear tests were performed on the coating using an AISI 52100 bearing steel counter face ball with a ball-on-disk configuration. A reduction in wear rate with an increase in friction coefficient due to three-body abrasion was reported.

Amino functionalized Ti_3_C_2_T_x_ nano-sheets were incorporated in water-borne epoxy composite coatings [72]. The functionalization of the filler aided in achieving its good dispersion in the resin and improved interfacial bonding with the substrate. Further, the stiffness of the coating matrix improved due to good mechanical properties of the filler. Consequently, the wear rate and friction coefficient reduced as indicated by the ball-on-plate dry sliding wear test results against steel ball. A further reduction was noticed with an increase in the content of functionalized Ti_3_C_2_T_x_ obtaining the best tribological and corrosion performance at 0.5 wt%.

Yu et al. [78] reinforced epoxy composite coatings with (0–1 wt%) functionalized CBN and HBN nano-fillers. The chemical modification of fillers with silane coupling agent (APTES) resulted in better dispersion in epoxy resin. Tribological performance of a series of composite coatings was evaluated by performing reciprocating ball-on-plate tribo-test against Si_3_N_4_ ball under dry and sea water conditions. A reduction in wear and friction was recorded with increasing content of either functionalized CBN and HBN nano-fillers (0–0.5 wt%) reaching an optimum value at 0.5 wt%. Increasing the filler content beyond 0.5 wt% increased the friction and wear due to agglomeration. The lubricating effect caused by sea water resulted in better friction and wear performance. The composite coating reinforced with functionalized CBN displayed the highest wear resistance due to higher hardness of CBN. The maximum reduction in friction was exhibited by coating reinforced with functionalized HBN. This was attributed to its lamellar structure promoting self-lubrication effect.

**Figure 3:**
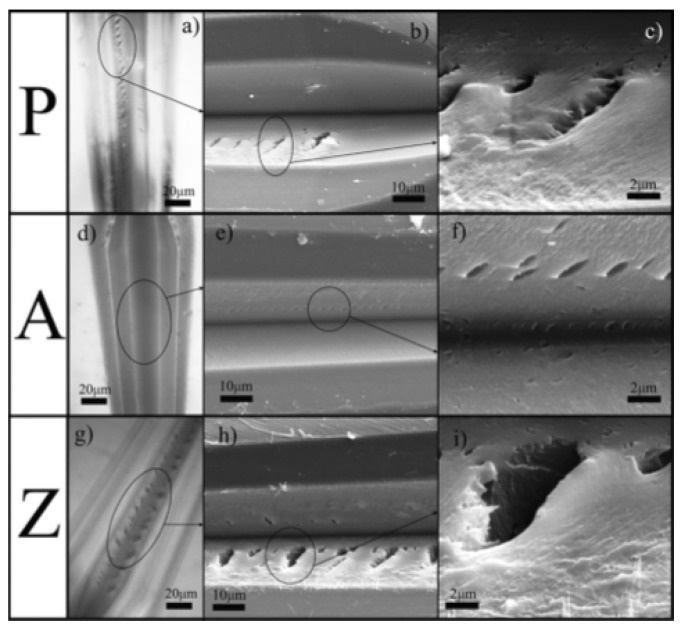
Optical microscopy (**a**,**d**,**g**) and scanning electronic microscopy(**b**,**c**,**e**,**f**,**h**,**i**) of scratches showing cracks for epoxy coating without filler (P), and nanocomposite coatings with Al_2_O_3_ (A) and ZnO (Z) Fillers [81].

Karasinski et al. [81] studied the effect of adding nano metal oxides (ZnO, Al_2_O_3_) to epoxy resin. The incorporation of ZnO nano-filler resulted in denser cross-linked network as a result of extended reaction owing to lower activation energy of the nanocomposite. Consequently, the nanocomposite exhibited a brittle behavior characterized with large and pointed cracks when subjected to scratch testing as shown in Figure 3. The toughening of epoxy nanocomposite coating with the incorporation of Al_2_O_3_ fillers was indicated by the scratch test results. Minimum influence of Al_2_O_3_ fillers on epoxy cure was credited to induce toughening mechanisms resulting in smaller cracks.

The usage of solid and liquid lubricant fillers was also found to be an effective means of tailoring tribological properties of epoxy coatings. Chen et al. [73] used niobium diselenide (NbSe_2_) nano-sheets as a solid lubricant in the fabrication of epoxy nanocomposite coatings. Ball-on-disk wear tests were performed against CGr15 steel ball to study the tribological performance. It was observed that a formation of transfer layer resulted in the reduction of friction coefficient by 80% with an increase in filler content from 0–20 wt%. Similarly, a 70% reduction in wear rate was reported at 10 wt% filler content. A slight increase in friction and wear at higher filler concentration was attributed to the agglomeration of excessive NbSe_2_ nano-sheets. While the friction coefficient decreased with increasing normal loads from 10 N to 30 N, the wear rate was found to increase. This indicated the unsuitability of the NbSe_2_/epoxy nanocomposite coating for heavy load applications.

In an another study, Li et al. [83] used encapsulated Tung oil as a liquid lubricant filler. The tung oil was selected due to its self-lubricating and self-healing properties. The epoxy coating was fabricated incorporating polysulfone microcapsules containing tung oil. Polysulfone was used as encapsulating material due to its high mechanical strength and outstanding physical, thermal, and chemical stability. A significant reduction in wear rate, friction coefficient, and corrosion rate was reported with modified coating. The self-lubricating effect and anti-corrosion performance was promoted by the formation of a transfer film.

In an attempt to synthesize cost effective epoxy based coatings, Akeem et al. [76] synthesized 1–20 wt% waste tire rubber particles (<70 µm) reinforced epoxy coatings. A reciprocating sliding wear test against stainless steel counter face was performed on a ball-on-disk tribometer. While the wear rate reduced in the range of 70–77% with the increment of reinforcement up to 10 wt%, the coefficient of friction reduced with increasing reinforcement content. At higher concentration of reinforcement, the coating was characterized with poor interfacial bonding, increased formation of voids and blends. Consequently, mechanical properties and wear performance deteriorated. Though wear rate was found to increase with increasing temperature, normal load, and sliding speed, normal load had a predominant effect.

**Figure 4:**
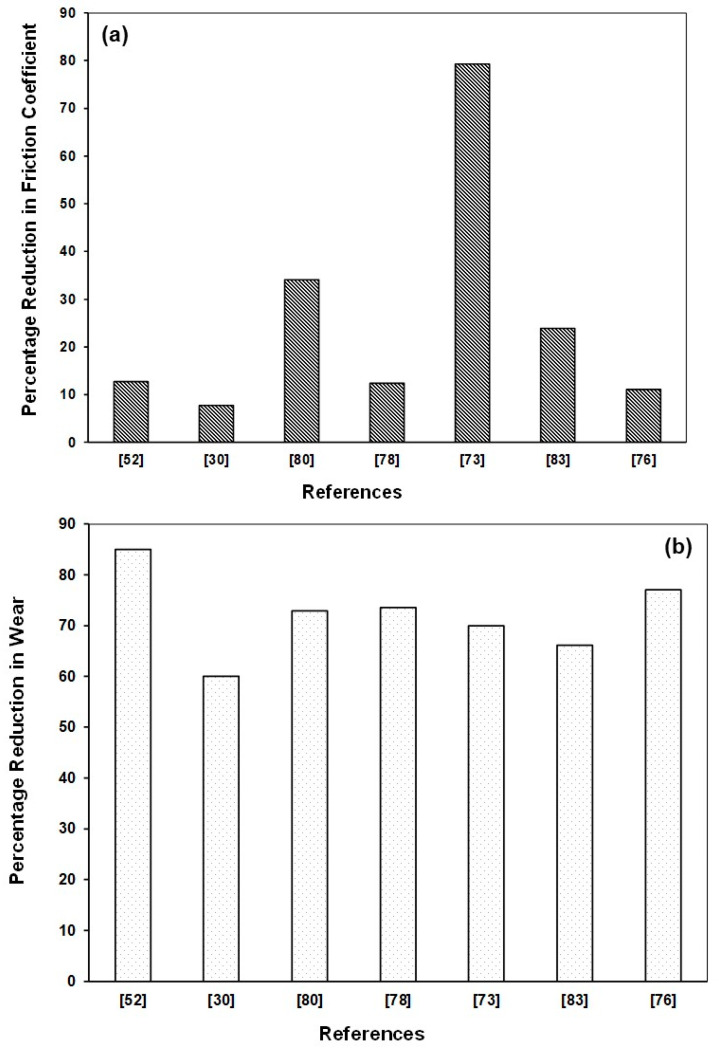
Comparison of effectiveness of LBE non-hybrid coatings in reducing (**a**) friction coefficient, (**b**) wear rate, under dry sliding conditions.

Based on the extensive literature review conducted above on the non-hybrid LBE composite coatings, Table 5 and Figure 4 have been generated to present a bird’s view of the effect of various nano-fillers added to the epoxy matrix on its coefficient of friction and wear. It can be clearly seen that among all the nanofillers, niobium diselenide (NbSe_2_) nano-sheets [73], a solid lubricant was effective in reducing both the COF and the wear, significantly. However, Tung Oil microcapsules results in the lowest wear rate among all the nano fillers. Graphene sheets were also effective in reducing the wear rate significantly by 85%, but with a lower reduction in COF of about 12.7%. Fillers such as HBN, Fullerenes are also effective in reducing the wear rates effectively with an appreciable reduction in COF. Hence, appropriate fillers are to be selected based upon the requirement, application and operating conditions.

#### 4.4.2. Hybrid LBE Composite Coatings

A comparative study of tribological properties of fluoropolymer modified epoxy coatings in combination with 1 μm–5 μm sized metallic fillers such as nickel (flakes form), aluminum (atomized), silver (irregular shaped) and zinc (irregular and spherical shaped) was carried out by Browstow et al. [67]. The bisphenol-A-based epoxy resin system was initially modified using 10 wt% FPEK fluoropolymer. Two types of curing agents namely, triethylenetetramine (TETA)—a low temperature curing agent and hexamethylenediamine (HMDA)—a high temperature curing agent, were used to cross-link the epoxy. The tribological properties of modified polymeric coatings were evaluated by performing ball-on-disk friction and wear tests against SS 302 grade stainless steel ball. Friction and wear rates were found to be dependent on the curing temperatures which were in turn dependent on the curing agent. During low temperature cure, the friction coefficient values were found to drop because of the migration of the fluoropolymer to the free surface of the epoxy owing to its lower surface energy. In case of high temperature cure, the cross-linking mechanism took preference over the phase separation process resulting in a mixture of epoxy and FPEK phases at the free surface resulting in higher frictional coefficient values. It was noted that aluminum fillers resulted in the least coefficient of friction and wear when cured at lower temperatures. This is due to their spherical morphology resulting in rolling friction between the contacting surfaces during the ball-on-disk test. It was also noted that irrespective of the curing agents used, addition of Zn resulted in an increase in friction and wear coefficient possibly because of the formation of a secondary phase of ZnO as described earlier. However, high temperature cure had an adverse effect on friction and wear properties of the epoxies modified with metallic powders.

**Figure 5:**
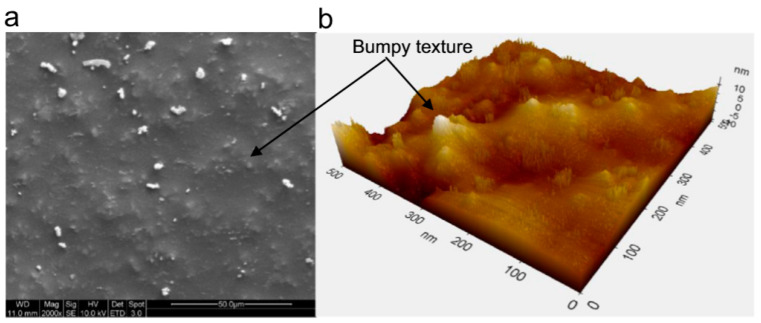
(**a**) FESEM image of unworn surface (**b**) AFM image of same surface showing bumpy texture [26].

Epoxy based composite and hybrid composite coatings with (5–30 wt%) graphite or (2.5–15 wt%) talc or mixed fillers were fabricated on glass substrates [26]. The optimum results were obtained in case of using mixed fillers at 15 wt% graphite + 15 wt% talc. Mechanical characterization at optimum composition showed increase in hardness by 2 times, and elastic modulus by 3 times. Ball-on-disk friction and wear tests were performed against Si_3_N_4_ ball up to the failure of coatings. No failure of coating was noticed under test conditions at the end of 4 × 10^5^ cycles when the tests were stopped owing to long duration. No scratch mark was noticed on counter face ball. Moreover, minimal amount of abrasive wear with wear debris consisting of mostly graphite was reported. Therefore, a reduction in specific wear rate by 99.98% and friction coefficient by 72.6% was reported for optimal composition. The outstanding tribological performance was attributed to the enhanced mechanical properties, lubricating effect of graphite together with micro-nano texturing (Figure 5). Such a texture in presence of lubricant has also resulted in the reduction of friction and wear by several orders of magnitude in other studies [87].

In order to improve the dispersion of Graphene oxide (GO) in epoxy resin, Zhao et al. [37] modified its surface by reducing it with dehydrated ethylenediamine. The reduced and modified GO along with PTFE nano-powders were used to hybridize epoxy coatings. The tribological performance of the coating was evaluated by performing reciprocating friction and wear tests against GCr15 steel ball. In comparison to pristine epoxy coating, the addition of 1 wt% modified and reduced GO and 10 wt% PTFE to the coating had resulted in the reduction of friction coefficient and wear rate by 70.6% and 20.9% respectively. The synergistic effect of the hybrid materials had not only prevented the formation of agglomerates (only up to 1 wt% modified and reduced GO), but also prevented the crack propagation. Additionally, the formation of transfer film at the interface resulted in improved tribological performance.

Yanjun ma et al. [53] hybridized the PTFE based epoxy composite coatings by in situ-synthesis of Ag_2_S nano-particles during curing process by pyrolysis reaction of silver dialkyldithiocarbamate. The smaller size and good dispersion of Ag_2_S particles was obtained. The effect of adding Ag_2_S, its particle size and size distribution on tribological performance of the coating was evaluated using linear reciprocal ball-on-disk tribometer. The existence of in-situ synthesized Ag_2_S particles had little effect on the average value of friction coefficient. A smaller size and a narrower size distribution of Ag_2_S particles resulted in reduction of wear rate by 26.41%, which was attributed to the formation of a protective tribo-film and an increase in the microhardness.

Petro Bandeira et al. [85] studied the effect of direct incorporation of ionic liquid alone, oxidized graphene nano-platelets (GNPox) alone and their combination on tribological performance of epoxy-PTFE based composite coatings. The ionic liquid used was 1-decyl-3-methylimidazoliumbis (trifluoromethylsulfonyl) imide [DMIM][NTf2] (DMIM). The incorporation of ionic liquid had no adverse effect on coating adhesion under test conditions. Crossed-cylinder type tribometer was used to evaluate friction and wear properties at two different loads (75 N and 150 N). The binder experienced plasticizing effect due to the lone use of ionic liquid inducing boundary lubrication. Greater coating stability was achieved with the lone usage of GNPox modifier indicating better interfacial bonding with epoxy. Combined usage of ionic liquid and GNPox with PTFE based epoxy coating had synergistic results. The boundary lubrication regime promoted the alignment of GNPox particles reducing their abrasive effect. Consequently, a reduction in friction coefficient was reported at higher loading condition.

To improve the durability of epoxy coatings, Han Yan et al. [80] synthesized the Ti_3_C_2_/graphene hybrid with wrapping structure through the bridge effect of polydopamine. Ball-on-plate wear tests against Al_2_O_3_ and Si_3_N_4_ balls under dry and 3.5 wt% NaCl solution were performed. In comparison to pristine epoxy, the friction coefficient and wear rate while sliding against Al_2_O_3_ reduced by 9.78% and 88.83% in dry sliding condition, 40.83% and 96.13% in aqueous media, respectively. A reduction in friction coefficient and wear rate while sliding against Si_3_N_4_ by 11.71% and 81.85% in dry condition and 31.95% and 98.80% in aqueous media respectively, was reported. The effective wear resistance of the hybrid composite coatings was also shown to promote the corrosion resistance.

The one-step hydrothermal method has emerged as common technique to synthesize solid lubricant nano-particles like ZnS, MoS_2_ on the surface of primary fillers such as reduced graphene oxide [45], CNTS [57], CNF [63], and HBN [72]. This helps to minimize the agglomeration of nano-particles and the stacking of primary fillers in the epoxy hybrid composite coatings. The friction and wear behavior of the coatings were evaluated by performing ball-on-disk sliding wear test against steel ball. The hybrid composite coating showed reduction in wear rate and friction coefficient compared to either the pristine epoxy coating or the epoxy composite coating filled separately with individual fillers. The outstanding tribological performance was credited to uniform dispersion of fillers, load bearing capabilities of CNTs, GO, CNF, HBN and the lubricating effect of ZnS, MoS_2_ and the formation of transfer film at the interface. The wear rate was found to increase with increasing applied load and sliding velocity in all the studies except in case of HBN/MoS_2_ [72] hybrid where it first increased and then decreased with sliding speed. An increase in the applied load increased friction coefficient in all the investigations [57,63,72] except in case of RGO/ZnS hybrid reinforced composite [45] where a reverse trend was observed. Moreover, sliding speed was reported to have mixed effect on friction coefficient. To further enhance the synergistic effect, epoxy coatings filled with CNT/GO/MoS_2_ ternary hybrid material synthesized by the same method was fabricated [62]. The coatings exhibited excellent tribological properties. While the wear rate of ternary hybrid epoxy composite coating reduced by 90%, the friction coefficient reduced by 95% in comparison to pristine epoxy coating. Though the wear rate and the friction coefficient were reported to increase with increasing applied load and sliding speed, they were lower in comparison to pristine epoxy coatings.

**Figure 6:**
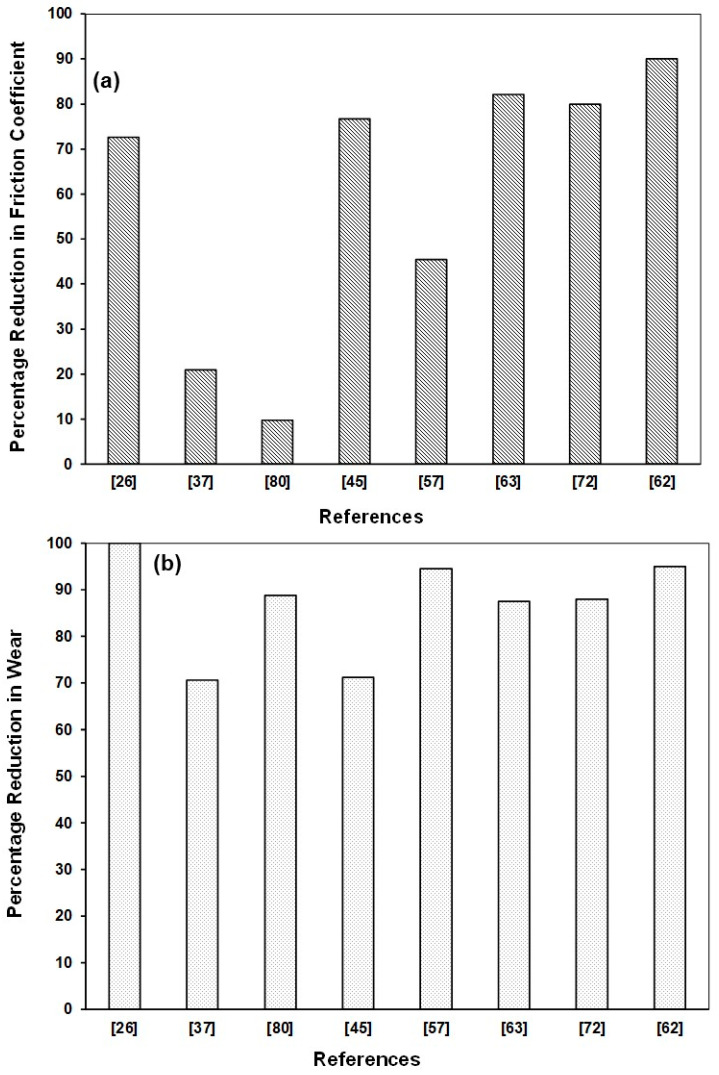
Comparison of effectiveness of LBE hybrid composite coatings in reducing (**a**) friction coefficient, (**b**) wear rate, under dry sliding conditions.

Table 6 and Figure 6 present the bird’s view of the effectiveness of the various combinations of nanofillers in reducing the COF and wear rates of an epoxy coating. It can be observed that, in general, a hybrid epoxy coating is very effective in reducing the COF and the wear rates significantly as compared to the epoxy composite coatings. Especially, the reduction in wear rates of the hybrid epoxy coatings is much higher as compared to that of the composite coatings. Among the different combinations of nanofillers, Graphite and Talc [26] showed the highest reduction in wear rate.

## 5. Fusion Bonded Epoxy (FBE) Coatings

Powder based epoxy coatings are commonly referred to as fusion bonded epoxy (FBE) systems. The absence of solvent in FBE system reduces environmental pollution, flammability and toxicity [88]. High temperature tolerant and corrosion resistant coatings with low-energy consumption, low-cost, and efficient application technology makes FBE systems attractive for industrial applications [89]. In particular, a major share of mainline pipe coatings in oil and gas industry are based on FBE technology with liquid epoxy or alternate systems such as visco-elastic tape wraps based on poly iso-butylene technology being limited only to girth weld areas [89]. Most of the work available in scientific literature is focused on liquid epoxy coatings or composite systems whereas literature pertaining to epoxy powder coatings which is equally important from an industrial point of view—is surprisingly scarce.

### 5.1. Fillers Used in FBE Coating Systems

Since the investigations pertaining to epoxy powder coatings are scarce, limited number of reinforcements were encountered in the review. Barletta et al. [88] used silica and alumina as curing catalysts; and titanium oxides as flowing promoters and UV stabilizers. M. Fernandez-Alvarez et al. [90] used SiO_2_ nano-particles as reinforcement in powder based epoxy coating because they are the most widely reported reinforcement to improve wear properties of organic powder coatings. Aluminosilicate nano-fillers were observed to reduce friction and wear of powder based epoxy coatings [89].

### 5.2. Dispersion Techniques Used in FBE Coating Systems

Conventionally, extrusion has been the mixing method for homogenizing organic powder coatings [90,91,92,93,94]. Grigoriev et al. [89] used twin-screw micro extruder for homogenizing epoxy based nanocomposite powders. Recently, hot mixing has emerged to be faster and cheaper alternative technique [90,95,96] which was also adopted by Fernandez-Alvarez et al. [90]. The dispersion techniques adopted by researchers are summarized in Table 7.

### 5.3. Coating Techniques Used in FBE Coating Systems

Electrostatic spray deposition is the most common method used for powder coating on the substrate. The powder is electrostatically charged by transporting it through a high voltage electrode. The charged powder cloud is sprayed on the grounded substrate by the combined action of electrostatic force and compressed air. The coated work piece is then subjected to curing at elevated temperatures commonly in convection oven to transform powder layer to a continuous film [88,89,90]. Prior to the application of coating, the substrate is generally grounded, polished and degreased. To create a highly conductive surface, the metallic substrates are sometimes coated with nickel-copper alloy [88]. If the substrate is electrically non-conductive such as polyamide [97], it is coated with a thin layer of copper. The coating techniques adopted by researchers along with different surface pre-treatments are summarized in Table 8.

### 5.4. Tribological Properties of FBE Coating Systems

Barletta et al. [88] evaluated the scratch and wear properties of powder based epoxy coatings at different stages of film curing process. Pin-on Disk dry sliding wear tests were performed against steel (100Cr) counter face under a normal load of 5 N and sliding speed of 0.2 m/s. Wear resistance increased with increasing baking temperature and time. Only the fully cured films could survive the entire wear test while the under cured films failed and could not survive the test duration of 6 min. The baking temperature and time greatly influenced the survival of the film in wear testing. Progressive load scratch tests were performed with loads ramping from 100 mN to 30 N at a rate of 5 N/min. A consistent improvement in scratch resistance with increasing baking temperature and time was reported. The under cured films (baked at 100 °C and 125 °C) experienced sudden increase in penetration and residual depth around a load of 20 N, indicating a faster onset of transition to film failure.

**Figure 7:**
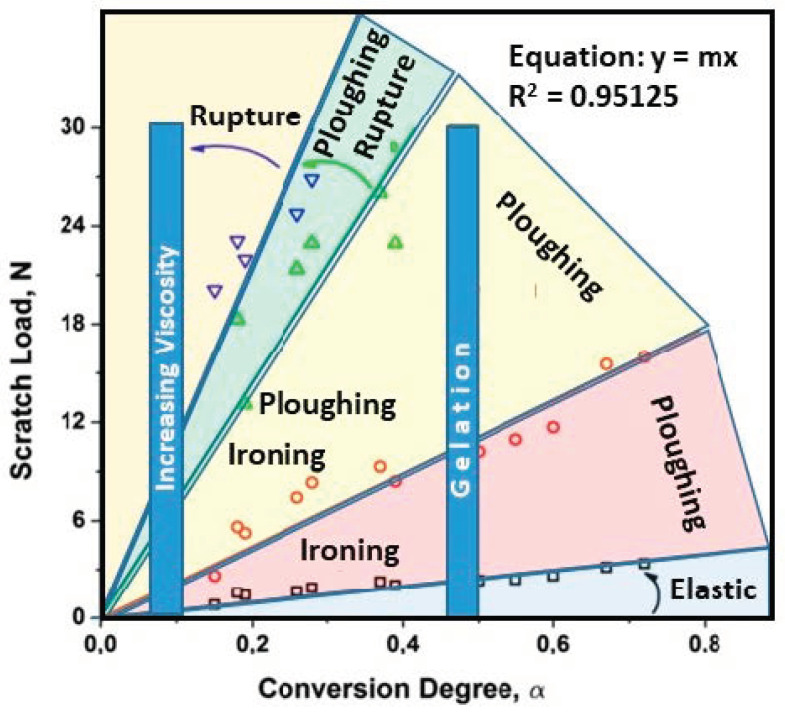
Scratch Map, reproduced from [88].

Using the obtained scratch and morphological data, a scratch map was created which presents the severity of damage experienced by the film as a function of applied scratch load and the degree of curing conversion (Figure 7).

The magnitude of damage associated with low, intermediate and high scratch loads was classified into elastic zone, ploughing zone at sufficient degree of conversion and Rupture zone at degree of conversion below 0.4–0.45 respectively. The embrittlement of the film beyond its induction period and well before gelation point (at low degree of curing conversion) subjects it to ironing at low loads, and fracture at intermediate and high loads, together with general ploughing. The film did not fracture over the applied load range (0–30 N) when it approaches gelation point (degree of curing conversion is greater than 0.4–0.45).

Grigoriev et al. [89] studied the effect of morphology and concentration of Aluminosilicate nano-fillers on tribological properties and mechanisms of epoxy powder composite coatings. The tubular and laminar forms of Aluminosilicates (0.5, 1, and 3 wt%) were separately mixed with epoxy resin, along with the hardening, pouring and degassing agents, by extrusion. The hardened composite was ground into powder with an average particle size of 125 µm. The powder coatings were electrostatically applied to steel substrate. Reciprocating dry sliding wear tests were performed against a steel ball using varying loads ranging from 100 to 1000 mN at a relative sliding velocity of 2.5 mm/s. An obvious reduction in friction and wear was observed with the incorporation of nano-fillers. The coating with flaky laminar nano-filler featured higher wear and friction coefficient compared to the coatings filled with tubular form of Aluminosilicate. This difference in friction and wear properties of composite coatings was attributed to the difference in their morphology and viscoelastic properties. The fractured surfaces of coatings were analyzed by SEM to understand viscoelastic deformation mechanisms. The epoxy coatings filled with laminar form of Aluminosilicate featured signs of lower rate of deformation propagation compared to that filled with tubular morphology. Friction coefficient and wear was also reported to reduce with increasing concentration of nano-fillers. Wear reduced with increasing concentration of nano-fillers due to reduction in adhesive interaction caused by the shielding effect of nanofillers at the friction interface. The friction coefficient was reported to reduce due to increase in elastic recovery at the frictional interface with increasing concentration of nano-fillers. This increase in elastic recovery induces elastic deformation reducing the friction forces over wide range of loads. Consequently, friction coefficient was seen to reduce with increasing filler concentration. Given the equal concentrations of nano-fillers, the tubular morphology of Aluminosilicate particles provides better trobological properties to the epoxy coatings.

Fernandez-Alvarez et al. [90] incorporated epoxy based powder coatings with varying weight percentages (1, 2 and 3 wt%) of SiO_2_ nano-particles. Novel hot mixing technique was used for homogeneous dispersion of nano-particles. The powder coating was applied on degreased carbon steel substrates by electrostatic gun. Scratch tests with 5 kg mass were performed on cured coatings (65 ± 5 µm). The ratio of delaminated lateral area to the scratch length was used as an indicator of scratch resistance. The smaller value of this ratio represents better scratch resistance. The increase in weight percentages of SiO_2_ nano-particles resulted in increasing scratch resistance. The scratch resistance was improved by 5 times with the incorporation of 3 wt% SiO_2_ nano-particles. The catalytic effect of nano-particles during curing process was credited for the improved scratch resistance. Reciprocating dry sliding wear tests were performed against stainless steel ball at 5 N. The width and depth of wear track was measured after the wear test to evaluate the wear resistance of the coatings. The measurements indicated that wear resistance improved and the friction coefficient reduced with increasing concentration of nano-particles. The increasing concentration of SiO_2_ nano-particles was observed to delay the onset of wear. A constant smooth friction coefficient curve for coatings containing 3 wt% SiO_2_ nano-particles indicate no onset of wear under the test conditions. Furthermore, the absence of wear track indicated almost no wear. Therefore, this coating was decided to be subjected to higher loads (5, 7.5, 10, 15, 18 N).

While the smaller loads of 5 and 7.5 N feature similar smooth friction curve with no sign of wear, the higher loads (10, 15, and 18 N) feature transition to abrasive wear regime. The time to transition is observed to reduce with increasing loads. The enhanced tribological performance of the powder coating was credited to the increased hardness and reduced plastic work due to the addition of nano SiO_2_ particles.

## 6. Conclusions/Future Trends

Even though epoxy coating systems have been extensively used in certain applications such as, fluid handling systems, their use in demanding tribological applications are known to be limited. This has been often attributed to their low load bearing capacity combined with the poor thermal stability under severe *p-v* regimes. Researchers in both academia and industry have tried to enhance the tribological properties of the pristine epoxy matrix using a combination of several types of micro/nano sized fillers to produce composites or hybrid nanocomposites. Hence, the current focused review tried to summarize the various efforts of different researchers, which shall act as an excellent starting point for people in this field to initiate their work. Based upon this extensive literature review we can summarize the following:

- Epoxy coatings are mainly classified into Liquid based (LBE) and Powder based (FBE) coatings. Various metallic, ceramic, polymeric, and carbon-based micro/nano fillers have been added to the epoxy matrix to improve the tribological performance of these coatings.
- It is observed that the dispersion of the nanofillers in the epoxy matrix is one of the most important factors affecting the overall properties of the developed coatings. Sonication was the most preferred technique used for dispersion, which is attributed to the high frequency ultrasonic waves, which promote uniform dispersion and de-agglomeration of fillers.
- Among the various coating techniques, wire bar coating emerges to be the most scalable technique for LBE coatings. However, it is to be noted, that the wire bar coatings were observed to have the slowest while the spin coatings had the fastest curing rate.
- Among the various nanofillers used to develop non-hybrid LBE composite coatings, niobium diselenide (NbSe_2_) nano-sheets, a solid lubricant found to be the most effective in reducing both the COF (79%) and the wear (70%) as compared to the pristine epoxy coating. Graphene sheets were also effective in reducing the wear rate significantly by 85%, but with a lower reduction in COF of about 12.7%.
- It is observed that, in general, a hybrid LBE coating is very effective in reducing the COF and the wear rates significantly as compared to the epoxy composite coatings. Among the different combinations of nanofillers, Graphite and Talc showed the highest reduction in wear rate (99%).
- Electrostatic spraying technique is found to be the most suitable for obtaining uniform thicknesses for the FBE coatings.

As observed from the literature, there is a lot of scope of development with respect to the epoxy coating systems in general and with respect to the FBE coating systems in particular. The industrial significance of the FBE coating systems makes it all the more necessary to explore the feasibility of developing new methods to improve their tribological performance.

## Figures and Tables

**Table 1:** Properties of common thermoset resins.

Property	Polyester	Vinylester	Epoxy
Specific gravity [11]	1.2	1.2	1.25
Tensile or compression strength (MPa) [12]	55	75	75
Young modulus in tension (MPa) [12]	3350	3350	3100
In-plane shear modulus (MPa) [12]	1350	1400	1500
Shear strength (MPa) [12]	approx. 50	approx. 65	approx. 80
Expansion coefficient (10^−6^ K^−1^) [12]	50–120	50–75	45–65
Cure shrinkage (%) [11]	5–12	5–10	1–5

**Table 2:** Properties of base and solidifier components used in epoxy formulation [6].

Generic	Type	General Properties
Base	Bisphenol-A	A reaction product of phenol and acetone; bisphenol-A is further reacted with epichlorohydrin to give DGEBA; possesses good abrasion, wear and impact resistance
	Bisphenol-F	A reaction product of phenol and formaldehyde; bisphenol-F is reacted with epichlorohydrin to give DGEBF; imparts better chemical resistance when compared to bisphenol-A
	Novolac	A reaction product of excess phenol and formaldehyde; imparts high temperature resistance but makes the epoxy matrix more brittle than bisphenol-A or bisphenol-F
Solidifier	Aliphatic amines	Imparts fast cure properties and solvent resistance at the expense of limited flexibility
	Polyamides and amidoamines	Improved flexibility and wetting-out characteristics are imparted to the epoxy matrix along with improved adhesion and water tolerance
	Cycloaliphatic amines	High flexibility and good impact resistance
	Aromatic amines	Lower heat resistance but good impact resistance

**Table 3:** Summary of various dispersion techniques used to uniformly disperse the nano-fillers in the epoxy matrix.

Ref	Filler	Filler Category	Class	Dispersion Technique
[84]	Graphene (0.1 wt%)	C-based	Non-hybrid	Epoxy Resin + graphene is dispersed in aprotic and protic ionic liquid by mechanical blending for 10 min + ultrasonic dispersion for 30 min.
[52]	Poly(2-butylaniline) functionalized Graphene	C-based	Non-hybrid	Poly(2-butylaniline) + Tetrahydrofuran (THF) sonicated for 30 min. Addition of epoxy with 10 min stirring. Removal of THF by rotary evaporation; addition of curing agent followed by blending at 4000 rpm for 5 min; room temperature degassing in vacuum oven.
[39]	Graphene (aryl diazonium Salt functionalized)	C-based	Non-hybrid	1 gm graphene dispersed in 30 mL distilled water through ultrasonication; Addition of dispersion to commercial coating base through mechanical stirring for 30 min.
[30]	Fullerene C60 Graphene	C-based	Non-Hybrid	Ultrasonic mixing of modified C60 or modified graphene with ethanol for 2 h; Addition of 6 gm epoxy with ultrasonic heating followed with removal of ethanol in oven; Addition of 1 gm solvent and 0.319 gm curing agent followed by 5 min stirring and degassing for 10 min.
[23]	MWCNTS	C-based	Non-hybrid	MWCNTs + Epoxy resin followed by 45 min sonication
[61]	CNT (pretreated)	C-based	Non-hybrid	Ball milling
[68]	Ferrite Particles post modified with Oleic acid	Metallic	Non-hybrid	0.6 gm Ferrite suspended in solution (60 mL isopropanol + 0.6 vol% Oleic acid) for 10 min; Sonication for 30 min followed by 2 h stirring at 60 °C; Particles infiltrated, cleaned in isopropanol and heated to 90 °C for 3 h; Addition of particles to epoxy resin followed by stirring and addition of curing agent.
[73]	Bulk niobium diselenide (NbSe_2_) Nano-sheets	Metallic	Non-hybrid	Epoxy Resin + (NbSe_2_ nano-sheets + acetone + ethanol) Room temperature stirring for 12 h by adding curing agent.
[76]	Waste Tire Rubber Particles	Polymeric	Non-hybrid	Epoxy Resin + (1–20 wt%) Micronized Tire Rubber Manual Stirring for 10 min; Addition of curing agent followed by manual stirring for 5 min.
[77]	Functionalized SiC particles	Ceramic based	Non-hybrid	2 h Mechanical stirring of (SiC + Epoxy resin) followed with ultrasonification for 2 h; Addition of hardener with slow stirring followed by heating at 60 °C for 2 h and 120 °C for 12 h.
[79]	Nano-SiO_2_ surface-capped with epoxide	Ceramic based	Non-hybrid	Dissolve 1 gm of epoxy resin in 10 mL of reagent Tetra hydrofuran; Add nano SiO_2_ surface modified with epoxide followed by 1h magnetic stirring.
[78]	Functionalized CBN Functionalized HBN	Ceramic based	Non-hybrid	Ultrasonic mixing of functionalized CBN or HBN (0–1 wt%) with ethanol for 2 h; Addition of 6 gm of Epoxy resin with ultrasonic heating followed with removal of residual ethanol in oven; Addition of 0.5 gm of xylene and 3 gm of curing agent followed by stirring and degassing for 10 min.
[80]	Amino-functionalized Ti_3_C_2_T_x_	Ceramic based	Non-hybrid	Ultrasonication of amino-functionalized Ti_3_C_2_T_x_ + 21 gm deionized water for 2 h; Addition of 20 gm water borne epoxy followed with 30 min high speed stirring; Addition of curing agent with 10 min stirring at 4000 rpm followed by room temperature degassing in vacuum oven.
[81]	ZnO nano-particles Al_2_O_3_ nano-particles	Ceramic	Non-Hybrid	Premix of epoxy resin, hardener, additives pellets and nano-fillers were fed to twin screw extruder at a screw speed of 490 rpm and 373 K.
[26]	Talc/Graphite	Mineral Silicate/C-based	Hybrid	Mix epoxy resin with Graphite and/or Talc using ultrasonic homogenizer for 25–30 min.
[41]	Ti_3_C_2_/graphene	Ceramic/C-based	Hybrid	Mix 0.5 wt% Ti_3_C_2_/graphene hybrid with water borne epoxy resin and stir for 0.5 h; Add deionized water to adjust viscosity; Add water borne curing agent and stir for 10 min at 4000 rpm followed with degassing in vacuum oven for 10 min at room temperature.
[37]	Reduced graphene oxide/PTFE	C-based/Polymeric	Hybrid	Mix 3 gm epoxy in 9 gm solvent; Add 1.5 gm curing agent followed with 30 min magnetic stirring; Add surface modified RGO (0–2 wt%) followed with 30 min magnetic stirring and 2 h bath sonication; Add PTFE nano powder followed with ball-milling for 6 h.
[45]	Reduced graphene oxide/Zinc sulfide	C-based/Metallic	Hybrid	Add Epoxy resin + reduced GO/ZnS hybrid material (mass ratio 3:1) to solvent (ethanol and acetone, 1:1) followed with 10 h stirring at RT. Addition of curing agent with 2 h stirring.
[85]	Oxidized graphene nanoplatelets/PTFE	C-based/Polymeric	Hybrid	Dispersion of 0.4 wt% GNPox and 4 wi% 1-decyl-3-methylimidazoliumbis(trifluoromethylsulfonyl)imide in N-methylpyrrolidone suspension (NMP) by ultrasonication for 150 min; Add 8 gm PTFE powder to 20 gm of suspension followed by stirring at 3000 rpm by rotor stator; Add 40 gm epoxy resin with stirring at 16,000 rpm by rotor-stator for 10 min; Mix 15.2 gm hardener.
[57]	CNTs/ZnS	C-based/Metallic	Hybrid	1 h Sonication of Epoxy + CNTs/ZnS + Acetone with stirring; Addition of curing agent with stirring and sonication for 15 min.
[63]	CNF/MoS_2_ core-shell hybrid	C-based/Metallic	Hybrid	1 h Sonication of Epoxy + CNF/MoS_2_ + Acetone with stirring; Addition of curing agent with stirring and sonication for 15 min.
[67]	10 wt% FPEK/ 25 wt% Ni, Al, Ag, Zn per 100 gm of epoxy	Polymer based/Metallic	Hybrid	FPEK dissolved in chloroform and epoxy resin added; calculated amounts of metallic powders added followed by vacuum treatment.
[53]	Ag_2_S/PTFE	Metallic/Polymeric	Hybrid	Disperse silver diethyldithiocarbamate precursor in solvents; Add binder resin and PTFE.
[72]	2D/2D HBN/ MoS_2_	Ceramic/Metallic	Hybrid	1 h Sonication of Epoxy + HBN/MoS_2_ + Acetone with stirring; Addition of curing agent with stirring and sonication for 15 min.
[82]	TiO_2_ Magnesium silicate	Ceramic/Mineral Silicate	Hybrid	Not defined
[62]	CNTs/GO/MoS_2_	C-based/C-based/Metallic	Hybrid	1 h Sonication of Epoxy + CNTs/GO/MoS_2_ + Acetone with stirring; Addition of curing agent with stirring and sonication for 15 min.

**Table 4:** Different types of coating techniques and pre-treatment techniques used by researchers.

Ref	Filler	Substrate	Pretreatment	Thickness	Coating Technique
[84]	Graphene	Mild Steel (AISI 1015)	Not Defined	21.2–48 µm	Spin Coating (1 min) and then oven cured for 2 h at 60 °C.
[52]	Poly(2-butylaniline) functionalized Graphene	Q235 Steel	Ultrasonicated in acetone; Nitrogen dried	20 ± 2 µm	Wire bar Coating, followed by room temperature curing for 3 days.
[39]	Graphene (aryl diazonium salt functionalized)	Steel	Not defined	15–25 µm	Spray gun followed with curing at (50–250 °C) for (0.5–1 h) at each temperature.
[30]	Fullerene C60, Graphene	Cast Iron	Not defined	30 µm 250 µm	Wire bar coated followed with room temperature curing for 7 days.
[23]	MWCNTs	AA2024-T3	Ultrasonicated in ethyl alcohol	2 µm: adhesion measurement 50 µm: tribology and corrosion	Draw down bar coated and cured at room temperature for 4 weeks.
[61]	CNT (1–5 wt%)	Mild Steel	Acetone cleansed	25–50 µm	Air sprayed followed with 30 min oven-curing 50–70 °C.
[68]	Ferrite Particles post modified with Oleic acid	Glass	Not defined	Not defined	Spin Coating followed by curing for 90 min at 90 °C.
[73]	Bulk niobium diselenide (NbSe_2_) Nano-sheets	Steel	Ultrasonicated in acetone for 10 min	Not defined	Air sprayed at 0.2 MPa nitrogen gas pressure; Solvent evaporation followed by 2 h curing at 100 °C.
[76]	Micronized Tire Rubber	Carbon Steel	Not Defined	600 ± 50 µm	Bar coating at a speed of 3 m/min, followed by room temperature curing for 24 h.
[77]	Functionalized SiC particles	Not defined	Not defined	2 mm	Not defined
[79]	Nano-SiO_2_ surface-capped with epoxide	Glass	Not defined	Not defined	Dropper coated followed by 2 h drying at 80 °C in vacuum oven.
[78]	Functionalized CBN Functionalized HBN	SS316	Not defined	300 ± 2 µm	Wire-bar coating followed with room temperature curing for 5 days.
[80]	Amino-functionalized Ti_3_C_2_T_x_	AA6082	Not defined	80 µm	Via Coater
[81]	ZnO, Al_2_O_3_ Nano-particles	Metallic	Not defined	100 ± 30 µm	Electrostatic Painting
[26]	Graphite/Talc	Glass Slides	Oxygen-plasma	45–50 µm	Spin Coated followed with prebaking at 70 °C and 95 °C for 5 and 10 min respectively; Exposed to UV rays for 50–60 s followed with baking for 80 °C for 5 min.
[41]	Ti_3_C_2_/graphene	AA6082	Not defined	80 µm	Spray painted followed with 24 h curing.
[37]	Reduced graphene oxide/ PTFE	Steel	Acetone cleansed	20 µm	Wire-bar coated followed with curing at 80 °C for 2 h.
[45]	Reduced graphene oxide/ zinc sulfide	Steel	Acetone and ethanol cleansed	Not defined	Air sprayed using a spray gun at 0.2 MPa nitrogen pressure followed by curing at 70 °C for 4 h.
[85]	Oxidized Graphene nanoplatelets/ PTFE	SAE52100 Steel	Air pyrolysis at 300 °C Al_2_O_3_ blasting	Not defined	Spray gun coating followed with 15 min curing at 250 °C in convection oven.
[57]	CNTs/ZnS	Steel	Ultrasonicated in alcohol (20 min)	Not defined	Air sprayed using a spray gun at 0.2 MPa argon pressure followed by curing at 100 °C for 2 h in vacuum oven.
[63]	CNF/MoS_2_ core-shell hybrid	Steel	Ultrasonicated in alcohol (20min)	50 µm	Air sprayed using a spray gun at 0.2 MPa argon pressure followed by curing at 100 °C for 2 h in vacuum oven.
[67]	10 wt% FPEK 25 wt% Ni, Al, Ag, Zn per 100 gm of epoxy	Mild steel (ASTM A366)	Lab conditions; No other pre-treatment	Not Defined	Brush
[53]	Ag_2_S/PTFE	SS AISI1045	Sand Blasted and Acetone cleansed	30 ± 5 µm	Air sprayed using a spray gun at 0.2M Pa air pressure followed by curing at 150 °C for 0.5 h and 240 °C for 2 h in drying oven.
[72]	HBN/MoS_2_ hybrid	Steel	Ultrasonicated in alcohol (20 min)	50 µm	Air sprayed using a spray gun at 0.2 MPa argon pressure followed by curing at 100 °C for 2 h in vacuum oven.
[82]	TiO_2_/ Magnesium silicate	Plain carbon/Stainless Steel	Epoxy phenolic primer applied	Not Defined	Brush coated
[62]	CNTs/GO/MoS_2_	Steel	Ultrasonicated in alcohol (20 min)	Not Defined	Air sprayed using a spray gun at 0.2 MPa argon pressure followed by curing at 100 °C for 2 h in vacuum oven.

**Table 5:** Comparison of tribological performance of non-hybrid LBE composite coatings with pristine epoxy coatings.

Filler	Filler Content (wt%)	Test Configuration	Test Condition	COF	% COF	Wear Rate (mm^3^/N-m)	% Wear	Ref
Graphene sheets	0.5	Ball-on-Plate	2 N-1 Hz-20 min-5 mm-316 steel Ball	0.48	12.7	7.10 × 10^−6^	85	[52]
CNT	1.5–2.5	Ball-on-Plate	0 to 1.2 N-1 Hz-13 mm-8 cycles	0.2	20	NA	NA	[61]
MWCNT	0.5	Ball-on-Disk	1 N-2 cm/s-12 m-Cr6 Steel Ball	0.27	18.18	NM	NM	[23]
Fullerene C60	0.5	Ball-on-Plate	3 3 N-1 Hz-20 min-5 mm-316 L steel ball	0.6	7.69	6.94 × 10^−4^	60	[30]
Fullerene C60	0.5	Ball-on-Plate ^l^	3 N-1 Hz-20 min-5 mm-316 L steel ball	0.25	45.65	5.56 × 10^−4^	68	[30]
Graphene ^d^	0.1	Ball-on-Disk	0.49 N-0.1 m/s-500 m-AISI 316L ball	0.17	70	NM	NM	[84]
Mn-Zn Ferrite ^s^	10	NA	300 to 500 g-0.01 mm/s-diamond tip	0.186	0	NA	NA	[68]
Ti_3_C_2_T_x_^a^ nano-sheets	0.5	Ball-on-Plate ^b^	3 N-2 Hz-30 min-5 mm-GCr steel ball	0.357	34.13	1.93 × 10^−4^	72.85	[80]
HBN ^a^	0.5	Ball-on-Plate ^b^	5 N-5 Hz-20 min-5 mm-Si_3_N_4_ ball	0.58	12.4	5.88 × 10^−4^	73.61	[78]
HBN ^a^	0.5	Ball-on-Plate ^b, l^	5 N-5 Hz-20 min-5 mm-Si_3_N_4_ ball	0.05	39.27	7.05 × 10^−4^	68.36	[78]
NbSe_2_	10	Ball-on-Disk ^b^	80 N-0.033 m/s-10 mm-10 min-GCr steel ball	0.07	79.23	1.48 × 10^−7^	70	[73]
Tung Oil-microcapsules	10	Pin-on-Disk	1 MPa-0.51 m/s-50 min	0.35	23.91	1.31 × 10^−13^	66.09	[83]
Rubber	5	Ball-on-Disk ^b^	20 N-10 Hz-5 mm	0.4	11.11	2.50 × 10^−4^	77	[76]

% COF—Percentage Reduction in Coefficient of Friction in comparison to pristine epoxy; % Wear—Percentage Reduction in wear rate in comparison to pristine epoxy; NA—Not Available; NM—Non Measurable; ^a^—functionalized; ^b^—reciprocating; ^d^—dispersed in apriotic liquid; ^s^—surface modified; ^l^—lubricated.

**Table 6:** Comparison of tribological performance of LBE hybrid composite coatings with pristine epoxy coatings.

Filler	Filler Content (wt%)	Test Configuration	Test Condition	COF	% COF	Wear Rate (mm^3^/N-m)	% Wear	Ref
Graphite Talc	15 15	Ball-on-Disk	2 N-0.28 m/s-Si_3_N_4_	0.2	72.60	1.30 × 10^−6^	99.98	[26]
RGO PTFE	1 10	Ball-on-Disk ^b^	5 N-4.2 Hz-30 min-5 mm-GCr15 Ball	0.139	20.9	15.21 × 10^−5 g^	70.6	[37]
PTFE Nano Ag_2_S	NA	Ball-on-Disk ^b^	10 N-11 cm/s-100 m-5 mm	0.074	0	5.60 × 10^−5^	26.41	[53]
DMIM Oxidized Graphene PTFE	4 0.4 20	Crossed-Cylinder	150 N-0.4 m/s-2 km-SAE52100 Steel	0.055	47.62	2.20 × 10^−6^	0	[85]
Ti_3_C_/_Graphene	0.5 *	Ball-on-Plate	5 N-2 Hz-30 min-4 mm-Al_2_O_3_	0.51	9.78	1.20 × 10^−4^	88.83	[80]
		Ball-on-Plate ^l^	5 N-2 Hz-30 min-4 mm-Al_2_O_3_	0.08	40.83	1.17 × 10^−6^	96.13	
RGO/ZnS	25 *	Ball-on-Disk	10 N-0.033 m/s-30 min-GCR Steel Ball	0.07	76.67	1.90 × 10^−6^	71.21	[45]
CNTs/ZnS	1.25 *	Ball-on-Disk	1.5 N-200 rpm-20 min-GCR Steel Ball	0.42	45.45	1.00 × 10^−4^	94.59	[57]
CNF/MoS_2_	1.25 *	Ball-on-Disk	4 N-200 rpm-20 min-440c SS Ball	0.075	82.14	8.60 × 10^−5^	87.52	[63]
h-BN/MoS_2_	1.5 *	Ball-on-Disk	4 N-200 rpm-20 min-440c SS ball	0.076	80	8.00 × 10^−5^	88	[72]
CNTs/GO/MoS_2_	1.25 *	Ball-on-Disk	3 N-200 rpm-20 min-440c SS ball	0.042	90	3.44 × 10^−5^	95	[62]

% COF—Percentage Reduction in Coefficient of Friction in comparison to pristine epoxy; % Wear—Percentage Reduction in wear rate in comparison to pristine epoxy; NA—Not Available; NM—Non Measurable; ^b^—reciprocating; ^l^—lubricated; ^g^—grams per min as the unit of wear rate; *—Weight % of hybrid filler.

**Table 7:** Summary of various dispersion techniques used to uniformly disperse the nano-fillers in the epoxy matrix.

Ref	Filler	Filler Category	Class	Dispersion Technique
[90]	NanoSilica	Ceramic based	non-hybrid	Hot mixing under dry conditions at 72 °C for 15 min at 40 rpm
[89]	Alumosilicate nano-particles	Mineral Silicate	non-hybrid	Mixed for 5 min in double screw micro-extruder at 200 rpm & 100 °C
[88]	SiO_2_, Al_2_O_3_, TiO_2_	Ceramic based	hybrid	Not Defined

**Table 8:** Different types of coating techniques and pre-treatment techniques used by researchers.

Ref	Filler	Substrate	Pretreatment	Thickness	Coating Technique
[90]	Nano Silica	Carbon Steel	Degreased	120 ± 20 µm	Electrostatically sprayed through a gun followed by oven curing for 15 min at 180 °C.
[89]	Alumosilicate nano-particles	Steel	Not Defined	80–100 µm	Electrostatic method followed by curing at 180 °C
[88]	SiO_2_, Al_2_O_3_, TiO_2_	Steel	Surface made electrically conductive by coating a thin layer of Ni-Cu alloy; Ultrasonicated in ethanol for 15 min	60–80 µm	Electrostatic Spray Deposition followed by heating in convection oven at different temperatures (100–200 °C) for different times (1–20 min)

## References

[1] Matthews A., Swift K.G. (1983). Intelligent knowledge-based systems for tribological coating selection. Thin Solid Films.

[2] Lockwood A. (2010). Evaluation of Corrosion and Wear of Non-Skid Deck Surfaces in Marine Environments. Master’s Thesis.

[3] Barbakadze K., Brostow W., Hnatchuk N., Hoyt Z., Lekishvili N. (2015). Tribology of novel antibiocorrosion coatings. Mater. Res. Innov..

[4] Silvestre C., López-Tendero M.J., Cruz-Yusta M., Baeza N., Guillem C., San Juan S., Lloris J.M., Tamayo E. (2009). Hybrid nanocomposite coatings for application in construction materials: Tribological study. Solid State Phenom..

[5] De la Isla A., Brostow W., Bujard B., Estevez M., Rogelio Rodriguez J., Vargas S., Castaño V.M. (2003). Nanohybrid scratch resistant coatings for teeth and bone viscoelasticity manifested in tribology. Mater. Res. Innov..

[6] Bobby S., Samad M.A. (2017). Enhancement of tribological performance of epoxy bulk composites and composite coatings using micro/nano fillers: A review. Polym. Adv. Technol..

[7] Marouf B., Bagheri R. (2017). Applications of Epoxy/Rubber Blends. Handbook of Epoxy Blends.

[8] Kumar V., Sinha S.K., Agarwal A.K. (2017). Tribological studies of epoxy composites with solid and liquid fillers. Tribol. Int..

[9] Chen H., Jacobs O., Wu W., Rüdiger G., Schädel B. (2007). Effect of dispersion method on tribological properties of carbon nanotube reinforced epoxy resin composites. Polym. Test..

[10] Wang Z., Yang M., Cheng Y., Liu J., Xiao B., Chen S., Huang J., Xie Q., Wu G., Wu H. (2019). Dielectric properties and thermal conductivity of epoxy composites using quantum-sized silver decorated core/shell structured alumina/polydopamine. Compos. Part A Appl. Sci. Manuf..

[11] Aranguren M.I., Reboredo M.M., Fakirov S., Bhattacharyya D. (2007). Plant-Based Reinforcements for Thermosets: Matrices, Processing, and Properties. Engineering Biopolymers: Homopolymers, Blends, and Composites.

[12] Ascione L., Caron J.-F., Godonou P., IJselmuijden K., Knippers J., Mottram J., Oppe M., Sorensen M., Taby J., Tromp L. (2016). Prospect for New Guidance in The Design of Frp.

[13] Durig J.D. (2000). Comparisons of epoxy technology for protective coatings and linings in wastewater facilities. J. Prot. Coat. Linings.

[14] Pulikkalparambil H., Siengchin S., Parameswaranpillai J. (2018). Corrosion protective self-healing epoxy resin coatings based on inhibitor and polymeric healing agents encapsulated in organic and inorganic micro and nanocontainers. Nano Struct. Nano Objects.

[15] Yan Z., Liu W., Gao N., Wang H., Su K. (2013). Synthesis and properties of a novel UV-cured fluorinated siloxane graft copolymer for improved surface, dielectric and tribological properties of epoxy acrylate coating. Appl. Surf. Sci..

[16] Kim J., Im H., Cho M.H. (2011). Tribological performance of fluorinated polyimide-based nanocomposite coatings reinforced with PMMA-grafted-MWCNT. Wear.

[17] Barbakadze K., Brostow W., Datashvili T., Hnatchuk N., Lekishvili N. (2018). Antibiocorrosive epoxy-based coatings with low friction and high scratch resistance. Wear.

[18] Liu D., Zhao W., Wu F., Cen Q., Zeng Z., Wu X., Xue Q. (2015). Effect of curing agent molecular structures on the tribological and corrosion behaviors of epoxy resin coatings. Colloids Surf. A Physicochem. Eng. Asp..

[19] Chen W.X., Tu J.P., Xu Z.D., Chen W.L., Zhang X.B., Cheng D.H. (2003). Tribological properties of Ni-P-multi-walled carbon nanotubes electroless composite coating. Mater. Lett..

[20] Wang C., Xue T., Dong B., Wang Z., Li H.L. (2008). Polystyrene-acrylonitrile-CNTs nanocomposites preparations and tribological behavior research. Wear.

[21] Zhang L.C., Zarudi I., Xiao K.Q. (2006). Novel behaviour of friction and wear of epoxy composites reinforced by carbon nanotubes. Wear.

[22] Li C., Chou T.W. (2003). Elastic moduli of multi-walled carbon nanotubes and the effect of van der Waals forces. Compos. Sci. Technol..

[23] Khun N.W., Troconis B.C.R., Frankel G.S. (2014). Effects of carbon nanotube content on adhesion strength and wear and corrosion resistance of epoxy composite coatings on AA2024-T3. Prog. Org. Coat..

[24] Zhang Z., Breidt C., Chang L., Haupert F., Friedrich K. (2004). Enhancement of the wear resistance of epoxy: Short carbon fibre, graphite, ptfe and nano-tio2. Compos. Part A Appl. Sci. Manuf..

[25] Pan G., Guo Q., Ding J., Zhang W., Wang X. (2010). Tribological behaviors of graphite/epoxy two-phase composite coatings. Tribol. Int..

[26] Katiyar J.K., Sinha S.K., Kumar A. (2016). Friction and wear durability study of epoxy-based polymer (SU-8) composite coatings with talc and graphite as fillers. Wear.

[27] Schniepp H.C., Li J.L., McAllister M.J., Sai H., Herrera-Alonson M., Adamson D.H., Prud’homme R.K., Car R., Seville D.A., Aksay I.A. (2006). Functionalized single graphene sheets derived from splitting graphite oxide. J. Phys. Chem. B.

[28] Lee C., Wei X., Kysar J.W., Hone J. (2008). Measurement of the Elastic Properties and Intrinsic Strength of Monolayer Graphene. Science.

[29] Qi Y., Liu J., Zhang J., Dong Y., Li Q. (2017). Wear Resistance Limited by Step Edge Failure: The Rise and Fall of Graphene as an Atomically Thin Lubricating Material. ACS Appl. Mater. Interfaces.

[30] Liu D., Zhao W., Liu S., Cen Q., Xue Q. (2016). Comparative tribological and corrosion resistance properties of epoxy composite coatings reinforced with functionalized fullerene C60 and graphene. Surf. Coat. Technol..

[31] Pan C., Kou K., Jia Q., Zhang Y., Wu G., Ji T. (2017). Improved thermal conductivity and dielectric properties of hBN/PTFE composites via surface treatment by silane coupling agent. Compos. Part B Eng..

[32] Pan C., Zhang L., Kou K., Zhang Y., Wu G. (2018). Investigation of the through-plane thermal conductivity of polymer composites with in-plane oriented hexagonal boron nitride. Int. J. Heat Mass Transf..

[33] Ghosh S., Calizo I., Teweldebrhan D., Pokatilov E.P., Nika D.L., Balandin A.A., Bao W., Miao F., Lau C.N. (2008). Extremely high thermal conductivity of graphene: Prospects for thermal management applications in nanoelectronic circuits. Appl. Phys. Lett..

[34] Zhu Y., Murali S., Cai W., Li X., Suk J.W., Potts J.R., Ruoff R.S. (2010). Graphene and graphene oxide: Synthesis, properties, and applications. Adv. Mater..

[35] Kasar A.K., Menezes P.L. (2018). Synthesis and recent advances in tribological applications of graphene. Int. J. Adv. Manuf. Technol..

[36] Wu Z.S., Ren W., Gao L., Liu B., Jiang C., Cheng H.M. (2009). Synthesis of high-quality graphene with a pre-determined number of layers. Carbon N. Y..

[37] Zhao B., Bai T. (2019). Improving the tribological performance of epoxy coatings by the synergistic effect between dehydrated ethylenediamine modified graphene and polytetrafluoroethylene. Carbon N. Y..

[38] Ramanathan T., Abdala A.A., Stankovich S., Dikin D.A., Herrera-Alonso M., Piner R.D., Adamson D.H., Schniepp H.C., Chen X., Ruoff R.S. (2008). Functionalized graphene sheets for polymer nanocomposites. Nat. Nanotechnol..

[39] Liu Y., Xia C., Zehri A., Ye L., Wang N., Zhmud B., Lu H., Liu J. (2019). Surface modification of graphene for use as a structural Fortifier in water-borne epoxy coatings. Coatings.

[40] Papageorgiou D.G., Kinloch I.A., Young R.J. (2017). Mechanical properties of graphene and graphene-based nanocomposites. Prog. Mater. Sci..

[41] Yan H., Zhang L., Li H., Fan X., Zhu M. (2020). Towards high-performance additive of Ti_3_C_2_/graphene hybrid with a novel wrapping structure in epoxy coating. Carbon N. Y..

[42] Kwon S., Ko J.H., Jeon K.J., Kim Y.H., Park J.Y. (2012). Enhanced nanoscale friction on fluorinated graphene. Nano Lett..

[43] Choi J.S., Kim J.S., Byun I.S., Lee D.H., Lee M.J., Park B.H., Lee C., Yoon D., Cheong H., Lee K.H. (2011). Friction anisotropy-driven domain imaging on exfoliated monolayer graphene. Science.

[44] Berman D., Erdemir A., Sumant A.V. (2013). Few layer graphene to reduce wear and friction on sliding steel surfaces. Carbon N. Y..

[45] Zhang S., Yang J., Chen B., Guo S., Li J., Li C. (2017). One-step hydrothermal synthesis of reduced graphene oxide/zinc sulfide hybrids for enhanced tribological properties of epoxy coatings. Surf. Coat. Technol..

[46] Berman D., Erdemir A., Sumant A.V. (2013). Reduced wear and friction enabled by graphene layers on sliding steel surfaces in dry nitrogen. Carbon N. Y..

[47] Llorente J., Román-Manso B., Miranzo P., Belmonte M. (2016). Tribological performance under dry sliding conditions of graphene/silicon carbide composites. J. Eur. Ceram. Soc..

[48] Jia Z., Chen T., Wang J., Ni J., Li H., Shao X. (2015). Synthesis, characterization and tribological properties of Cu/reduced graphene oxide composites. Tribol. Int..

[49] Ko J.H., Kwon S., Byun I.S., Choi J.S., Park B.H., Kim Y.H., Park J.Y. (2013). Nanotribological properties of fluorinated, hydrogenated, and oxidized graphenes. Tribol. Lett..

[50] Park J.Y., Kwon S., Kim J.H. (2014). Nanomechanical and Charge Transport Properties of Two-Dimensional Atomic Sheets. Adv. Mater. Interfaces.

[51] Lee H., Son N., Jeong H.Y., Kim T.G., Bang G.S., Kim J.Y., Shim G.W., Goddeti K.C., Kim J.H., Kim N. (2016). Friction and conductance imaging of sp2- and sp3-hybridized subdomains on single-layer graphene oxide. Nanoscale.

[52] Chen C., Qiu S., Cui M., Qin S., Yan G., Zhao H., Wang L., Xue Q. (2017). Achieving high performance corrosion and wear resistant epoxy coatings via incorporation of noncovalent functionalized graphene. Carbon N. Y..

[53] Ma Y., Wan H., Ye Y., Chen L., Li H., Zhou H., Chen J. (2020). In-situ synthesis of size-tunable silver sulfide nanoparticles to improve tribological properties of the polytetrafluoroethylene-based nanocomposite lubricating coatings. Tribol. Int..

[54] Ding Y., Zhou Y., Nie W., Chen P. (2015). MoS 2-GO nanocomposites synthesized via a hydrothermal hydrogel method for solar light photocatalytic degradation of methylene blue. Appl. Surf. Sci..

[55] Stankovich S., Dikin D.A., Dommett G.H.B., Kohlhaas K.M., Zimney E.J., Stach E.A., Piner R.D., Nguyen S.B.T., Ruoff R.S. (2006). Graphene-based composite materials. Nature.

[56] Yu X., Zhang W., Zhang P., Su Z. (2017). Fabrication technologies and sensing applications of graphene-based composite films: Advances and challenges. Biosens. Bioelectron..

[57] Li X., Chen B., Jia Y., Li X., Yang J., Li C., Yan F. (2018). Enhanced tribological properties of epoxy-based lubricating coatings using carbon nanotubes-ZnS hybrid. Surf. Coat. Technol..

[58] Yan L., Wang H., Wang C., Sun L., Liu D., Zhu Y. (2013). Friction and wear properties of aligned carbon nanotubes reinforced epoxy composites under water lubricated condition. Wear.

[59] Lee C.J., Park J., Kang S.Y., Lee J.H. (2000). Growth and field electron emission of vertically aligned multiwalled carbon nanotubes. Chem. Phys. Lett..

[60] Treacy M.M.J., Ebbesen T.W., Gibson J.M. (1996). Exceptionally high Young’s modulus observed for individual carbon nanotubes. Nature.

[61] Le H.R., Howson A., Ramanauskas M., Williams J.A. (2012). Tribological characterisation of air-sprayed epoxy-CNT nanocomposite coatings. Tribol. Lett..

[62] Chen B., Li X., Jia Y., Xu L., Liang H., Li X., Yang J., Li C., Yan F. (2018). Fabrication of ternary hybrid of carbon nanotubes/graphene oxide/MoS_2_ and its enhancement on the tribological properties of epoxy composite coatings. Compos. Part A Appl. Sci. Manuf..

[63] Chen B., Jia Y., Zhang M., Liang H., Li X., Yang J., Yan F., Li C. (2019). Tribological properties of epoxy lubricating composite coatings reinforced with core-shell structure of CNF/MoS_2_ hybrid. Compos. Part A Appl. Sci. Manuf..

[64] Bafekrpour E., Yang C., Natali M., Fox B. (2013). Functionally graded carbon nanofiber/phenolic nanocomposites and their mechanical properties. Compos. Part A Appl. Sci. Manuf..

[65] Yuan H., Yang S., Liu X., Wang Z., Ma L., Hou K., Yang Z., Wang J. (2017). Polyimide-based lubricating coatings synergistically enhanced by MoS_2_@HCNF hybrid. Compos. Part A Appl. Sci. Manuf..

[66] Ravindran A.R., Ladani R.B., Wu S., Kinloch A.J., Wang C.H., Mouritz A.P. (2018). Multi-scale toughening of epoxy composites via electric field alignment of carbon nanofibres and short carbon fibres. Compos. Sci. Technol..

[67] Brostow W., Dutta M., Rusek P. (2010). Modified epoxy coatings on mild steel: Tribology and surface energy. Eur. Polym. J..

[68] Wang W., Zang C., Jiao Q. (2015). Wear-resistant and electromagnetic absorbing behaviors of oleic acid post-modified ferrite-filled epoxy resin composite coating. J. Magn. Magn. Mater..

[69] Korolev V.V., Balmasova O.V., Ramazanova A.G. (2009). The sorption isotherms of oleic, linoleic, and linolenic acids from solutions in cyclohexane and heptane on magnetite. Russ. J. Phys. Chem. A.

[70] Dai Q., Lam M., Swanson S., Yu R.H.R., Milliron D.J., Topuria T., Jubert P.O., Nelson A. (2010). Monodisperse cobalt ferrite nanomagnets with uniform silica coatings. Langmuir.

[71] Shen L., Stachowiak A., Fateen S.E.K., Laibinis P.E., Hatton T.A. (2001). Structure of alkanoic acid stabilized magnetic fluids. A small-angle neutron and light scattering analysis. Langmuir.

[72] Chen B., Zhang M., Li X., Dong Z., Jia Y., Li C. (2020). Tribological properties of epoxy-based self-lubricating composite coating enhanced by 2D/2D h-BN/MoS_2_ hybrid. Prog. Org. Coat..

[73] Chen J., Yang J., Chen B., Liu S., Dong J., Li C. (2016). Large-scale synthesis of NbSe_2_ nanosheets and their use as nanofillers for improving the tribological properties of epoxy coatings. Surf. Coat. Technol..

[74] Han H.S., Tan K.L., Kang E.T. (2000). Fluorination of epoxy surfaces by a physical method. J. Appl. Polym. Sci..

[75] Brostow W., E Cassidy P., E Hagg H., Jaklewicz M., E Montemartini P. (2001). Fluoropolymer addition to an epoxy: Phase inversion and tribological properties. Polymer.

[76] Adesina A.Y., Zainelabdeen I.H., Dalhat M.A., Mohammed A.S., Sorour A.A., Al-Badou F.A. (2020). Influence of micronized waste tire rubber on the mechanical and tribological properties of epoxy composite coatings. Tribol. Int..

[77] Hao Y., Zhou X., Shao J., Zhu Y. (2019). The influence of multiple fillers on friction and wear behavior of epoxy composite coatings. Surf. Coat. Technol..

[78] Yu J., Zhao W., Wu Y., Wang D., Feng R. (2018). Tribological properties of epoxy composite coatings reinforced with functionalized C-BN and H-BN nanofillers. Appl. Surf. Sci..

[79] Kang Y., Chen X., Song S., Yu L., Zhang P. (2012). Friction and wear behavior of nanosilica-filled epoxy resin composite coatings. Appl. Surf. Sci..

[80] Yan H., Cai M., Li W., Fan X., Zhu M. (2020). Amino-functionalized Ti_3_C_2_T_x_ with anti-corrosive/wear function for waterborne epoxy coating. J. Mater. Sci. Technol..

[81] Karasinski E.N., Da Luz M.G., Lepienski C.M., Coelho L.A.F. (2013). Nanostructured coating based on epoxy/metal oxides: Kinetic curing and mechanical properties. Thermochim. Acta.

[82] Correa C.E., García G.L., García A.N., Bejarano W., Guzmán A.A., Toro A. (2011). Wear mechanisms of epoxy-based composite coatings submitted to cavitation. Wear.

[83] Li H., Cui Y., Wang H., Zhu Y., Wang B. (2017). Preparation and application of polysulfone microcapsules containing tung oil in self-healing and self-lubricating epoxy coating. Colloids Surf. A Physicochem. Eng. Asp..

[84] Avilés M.D., Jiménez A.E., Saurín N., Carrión F.J., Sanes J., Bermúdez M.D. (2020). Tribological characterization of epoxy coatings modified with ionic liquids and graphene. Tribol. Int..

[85] Bandeira P., Monteiro J., Baptista A.M., Magalhães F.D. (2016). Influence of oxidized graphene nanoplatelets and [DMIM][NTf2] ionic liquid on the tribological performance of an epoxy-PTFE coating. Tribol. Int..

[86] Kumar V., Sinha S.K., Agarwal A.K. (2015). Tribological studies of epoxy and its composite coatings on steel in dry and lubricated sliding. Tribol. Mater. Surf. Interfaces.

[87] Satyanarayana N., Lau K.H., Sinha S.K. (2008). Nanolubrication of poly(methyl methacrylate) films on Si for microelectromechanical systems applications. Appl. Phys. Lett..

[88] Barletta M., Lusvarghi L., Mantini F.P., Rubino G. (2007). Epoxy-based thermosetting powder coatings: Surface appearance, scratch adhesion and wear resistance. Surf. Coat. Technol..

[89] Grigoriev A.Y., Vaganov G.V., Yudin V.E., Myshkin N.K., Kovaleva I.N., Gofman I.V., Mashlyakovskii L.N., Tsarenko I.V. (2012). Friction and wear of powder coatings of epoxy composites with alumosilicate nanoparticles. J. Frict. Wear.

[90] Fernández-Álvarez M., Velasco F., Bautista A. (2020). Epoxy powder coatings hot mixed with nanoparticles to improve their abrasive wear. Wear.

[91] Deflorian F., Rossi S., Fedel M., Ecco L.G., Paganica R., Bastarolo M. (2014). Study of the effect of corrosion inhibitors on powder coatings applied on steel. Prog. Org. Coat..

[92] Shi Q., Huang W., Zhang Y., Zhang Y., Xu Y., Guo G. (2012). Curing of polyester powder coating modified with rutile nano-sized titanium dioxide studied by DSC and real-time FT-IR. J. Therm. Anal. Calorim..

[93] Catarina G.A.S., Borsoi C., Romanzini D., Piazza D., Kunst S.R., Scienza L.C., Zattera A.J. (2017). Development of acrylic-based powder coatings with incorporation of montmorillonite clays. J. Appl. Polym. Sci..

[94] Gioia C., Minesso A., Cavalieri R., Marchese P., Celli A., Colonna M. (2015). Powder coatings for indoor applications from renewable resources and recycled polymers. J. Coat. Technol. Res..

[95] Sharifi M., Ebrahimi M., Jafarifard S. (2017). Preparation and characterization of a high performance powder coating based on epoxy/clay nanocomposite. Prog. Org. Coat..

[96] Fernández-Álvarez M., Velasco F., Bautista A., Abenojar J. (2020). Effect of silica nanoparticles on the curing kinetics and erosion wear of an epoxy powder coating. J. Mater. Res. Technol..

[97] Barletta M., Gisario A., Tagliaferri V. (2006). Electrostatic spray deposition (ESD) of polymeric powders on thermoplastic (PA66) substrate. Surf. Coat. Technol..

